# Nanotherapeutic delivery of antibiotic cocktail enhances intra-macrophage killing of *Mycobacterium marinum*

**DOI:** 10.3389/frabi.2023.1162941

**Published:** 2023-07-17

**Authors:** Andrea M. Binnebose, Adam S. Mullis, Shannon L. Haughney, Balaji Narasimhan, Bryan H. Bellaire

**Affiliations:** ^1^ Department of Veterinary Microbiology and Preventive Medicine, Iowa State University, Ames, IA, United States; ^2^ Department of Interdepartmental Microbiology Graduate Program, Iowa State University, Ames, IA, United States; ^3^ Cargill Animal Nutrition, Elk River, MN, United States; ^4^ Department of Chemical and Biological Engineering, Iowa State University, Ames, IA, United States

**Keywords:** *Mycobacterium*, nanotherapeutic, antimicrobial delivery, antibiotic cocktail, intracellular delivery drug release

## Abstract

*Mycobacterium marinum* is a waterborne pathogen responsible for tuberculosis-like infections in cold-blooded animals and is an opportunistic pathogen in humans. *M. marinum* is the closest genetic relative of the *Mycobacterium tuberculosis* complex and is a reliable surrogate for drug susceptibility testing. We synthesized and evaluated two nanoparticle (NP) formulations for compatibility with rifampicin, isoniazid, pyrazinamide, and ethambutol (PIRE), the front-line antimycobacterial drugs used in combination against active tuberculosis infections. Improved *in vitro* antimicrobial activity was observed with encapsulated rifampicin alone or in a cocktail of drugs formulated through co-encapsulation in amphiphilic polyanhydride NPs. Broth antimicrobial testing revealed that the encapsulation of PIRE in NP resulted in a significant increase in antimicrobial activity, with the benefit over soluble formulations at biologically relevant concentrations ranging from >10 to >3,000 fold. *M. marinum-*infected human macrophages treated with NP-PIRE were cleared of viable bacteria in 48 h following a single treatment, representing a >4 log reduction in colony-forming units and a >2,000-fold increase in antimicrobial activity. The amphiphilic polyanhydride nanoparticles demonstrated the ability to co-encapsulate PIRE antibiotics and enhance their antimicrobial activity against *M. marinum* in infected macrophages in culture and *in vitro*. These data suggest that polyanhydride nanoparticles are a promising nanotherapeutic for combatting *Mycobacterium* infections through improved intracellular targeting of encapsulated antibiotics.

## Introduction

*Mycobacterium tuberculosis* is the ninth leading cause of death worldwide and the leading cause of death from a single infectious agent worldwide (Klopper et al., 2013). Almost 10% of new cases reported are resistant to the front-line antibiotic rifampicin, and 5% of new cases are multi-drug resistant (MDR) to at least rifampicin (RIF) and isoniazid (INH) (WHO, 2023). Clinical features of tuberculosis include multi-stage primary infection, latency, and reactivation (Saravanan et al., 2018). For years, efforts have been made to reduce the side-effects of treatment and promoting resistant and persistent phenotypes by understanding the mechanisms of the organisms’ pathogenesis (Forrellad et al., 2013; Namouchi et al., 2017). Researchers have used multiple species of mycobacteria to study the pathogenesis of tuberculosis and resistance mechanisms, including *M*. *smegmatis*, *M. bovis*, and *M. marinum*. Of these models, the non-tuberculous *M. marinum* is the closest genetic relative of the *M. tuberculosis* complex and causes tuberculosis-like granuloma formation in ectotherms (Ouertatani-Sakouhi et al., 2017). Eighty-five percent of *M. marinum* loci encoding recognized virulence genes have homologous genes in *M. tuberculosis* (El-Etr et al., 2004). *M. marinum* is widely used as a model to study mycobacterial infections and screen antimycobacterial compounds owing to its similar pathogenicity, ability to cause granuloma formation, and latency profile to *Mycobacterium tuberculosis* (El-Etr et al., 2004; Kenyon et al., 2017; Lienard and Carlsson, 2017).

Non-tuberculous mycobacteria (NTM), including *M. marinum*, cause several different types of infections, including respiratory, cutaneous, and systemic (Patel et al., 2014), many of which are multi-drug resistant (Griffith et al., 2007; Cheung et al., 2012)*. M. marinum* is an atypical bacillus resistant to anti-tubercular medications isoniazid, pyrazinamide (PZA), and para-aminosalicylic acid and shows intermediate sensitivity to streptomycin (Rallis and Koumantaki-Mathioudaki, 2007). Unlike the expected slow growth of 3–6 weeks for *M. tuberculosis*, *M. marinum* grows within 1–2 weeks, reducing the time required for *in vitro* drug analysis. *M. marinum* shares the complex cellular wall (Rodrigues et al., 2011; Verschoor et al., 2012) that pyrazinamide and isoniazid can penetrate only in prodrug (metabolically activated) form in the treatment of tuberculosis (Bhat et al., 2017). Most soluble-free drugs are internalized by macrophages (Fischer et al., 1996) and stored within lysosomes, where the bioactivity of the drugs is low (Sakhrani and Padh, 2013). *Mycobacterium* species’ ability to prevent the enzymatic conversion of these prodrug antibiotics (Anguru et al., 2017) and their slow growth rate (Bacon et al., 2014) reduces the activity of the drug. Therefore, limited intracellular activity against non-replicating bacteria is common. Because the therapeutic efficacy of most anti-tubercular drugs is well recognized, inefficient delivery could result in a low therapeutic index, causing persistent and latent infection (Hussain et al., 2019).

One approach to improving the efficacy of single-drug therapy has been to design treatment regimens based on the pharmacodynamics of antibiotics through controlled release and improving intracellular targeting (Hussain et al., 2019). Current treatments for *M. marinum* infections include streptomycin and gentamicin, with doxycycline as an emerging alternative therapy (Medel-Plaza and Esteban, 2023). Resistance of *Mycobacterium* species to individual drugs is common, with some cases of tuberculosis in humans requiring lengthy treatment regimens involving potent multi-drug treatments (Hussain et al., 2019; Bendre et al., 2021). We describe developing a drug delivery platform that can overcome *Mycobacterium* species’ inherent multi-facilitated, drug-resistant nature using amphiphilic polyanhydride nanoparticles as a drug delivery system. Polyanhydride nanoparticles are internalized by infected macrophages, allowing them to release anti-tubercular drugs intracellularly.

This work analyzes the effect of amphiphilic nanoparticle chemistry on the intracellular pathogen *M. marinum*. Previous research has demonstrated the increased efficacy and tissue distribution of drugs delivered by the encapsulation of antibiotics into polyanhydride nanoparticles against intracellular bacterial pathogens (Lueth et al., 2019). Using polymer and copolymer combinations of nanoparticles, a benefit of encapsulating drugs into nanoparticles was observed; encapsulation resulted in increased antimicrobial activity through enabling the sustained release of the drugs at low concentrations (Binnebose et al., 2015). Varying the polymer and copolymer combinations of the nanoparticles contributed to the antimicrobial activity of different drug combinations (Mullis et al., 2019). In this publication, we share the results of nanoparticles designed for the first-line anti-tubercular drugs rifampicin, isoniazid, and pyrazinamide to improve their antimicrobial activity against intracellular *M. marinum.*

## Materials and methods

### Chemical and reagents

The nanoparticle polymers used were 1,6-bis(pcarboxyphenoxy)hexane (CPH) and 1,8-bis(p-carboxyphenoxy)-3,6-dioxaoctane (CPTEG). Synthesized CPH and CPTEG monomers, polyanhydride polymers, 20:80 CPH : SA, 20:80 CPTEG : CPH, and 50:50 CPTEG : CPH were purchased from Fisher Scientific (Fairlawn, NJ), along with the rifampicin-, isoniazid-, pyrazinamide-, and ethambutol-loaded polyanhydride nanoparticles; acetic acid; acetic anhydride; acetone; acetonitrile; chloroform; dimethyl formamide; ethyl ether; hexane; methylene chloride; pentane; petroleum ether; potassium carbonate; sodium hydroxide; sulfuric acid; and toluene. The chemicals 1,6-dibromohexane, 1-methyl-2-pyrrolidinone, hydroxybenzoic acid, N,N-dimethylacetamide, sebacic acid, and tri-ethylene glycol were obtained from Sigma Aldrich (St. Louis, MO). The chemical 4-p-fluorobenzonitrile was purchased from Apollo Scientific (Cheshire, UK). For the ^1^H NMR analysis, deuterated chloroform and deuterated dimethyl sulfoxide were purchased from Cambridge Isotope Laboratories (Andover, MA). The fluorescent dye Rhodamine B was purchased from Sigma Life Science (St. Louis, MO). Rifampicin, isoniazid, pyrazinamide, and ethambutol were also purchased from Sigma Life Science (St. Louis, MO).

### Nanoparticle fabrication

The monomers used, 1,6-bis(p-carboxy phenoxy hexane) (CPH) and 1,8-bis(p-carboxyphenoxy)-3,6-dioxaoctane (CPTEG), were synthesized as described elsewhere (Torres et al., 2006). A molar ratio of 20:80 was employed for copolymers of CPTEG and CPH, and CPH and SA, and they were synthesized through melt condensation polymerization, as previously described in detail (Domb et al., 1993; Kipper et al., 2002; Torres et al., 2006). Molecular weight was confirmed using ^1^H NMR.

Rifampicin-, isoniazid-, pyrazinamide-, and ethambutol-loaded nanoparticles of 20:80 CPH : SA and 28:80 CPTEG : CPH were constructed through solid/oil/oil nanoprecipitation, as previously described (Ulery et al., 2011; Haughney et al., 2013; Huntimer et al., 2013). In summary, drugs were homogenized by sonication with copolymer solutions in the solvent methylene chloride. The copolymer–antibiotic mixture was then rapidly poured into a bath of anti-solvent pentane held at −40°C at an anti-solvent to solvent ratio of 1:80 and 1:150 for CPH : SA and CPTEG : CPH, respectively. Following the rapid precipitation of nanoparticles in the solvent–anti-solvent mixture, the resulting drug-loaded nanoparticles were collected using vacuum filtration and characterized by size and morphology using scanning electron microscopy (SEM; FEI Quanta SEM, Hillsboro, OR). Nanoparticles were stored at −20°C and prepared by sonicating 10 μg/mL phosphate-buffered saline (PBS) to generate fresh working stocks for each experiment.

### Bacterial strains and growth conditions

The *M. marinum* Aronson strain (ATCC 927) was propagated at 30°C either in Middlebrook 7H9 broth (Difco) containing 10% oleic acid-albumin-dextrose-catalase (Difco) and 0.05% Tween 80 (Sigma) or on Middlebrook 7H10 agar containing 10% oleic acid-albumin-dextrose-catalase. Frozen stocks were prepared by growing bacteria in 7H9 broth to mid-log phase (equivalent to an optical density at 600 nm [OD600] of ∼0.5), collecting cells by centrifugation, washing once with phosphate-buffered saline containing 0.05% Tween 80 (PBST), adding glycerol to a concentration of 15%, and storing in aliquots at −80°C. Bacterial stocks were titered by plating PBST-diluted bacterial suspensions on 7H10 agar and scoring colonies after 1 week at 30°C. The recombinant strain of *M. marinum* expressing green fluorescent protein (*M. marinum*- GFP) bears an integrative plasmid (pGFPHYG2). The pGFPHYG2 was a gift from Lalita Ramakrishnan (Addgene plasmid #30173), and the electroporation and selection of transformants were carried out as previously described (Parish and Stoker, 1995). Drug solutions were prepared at concentrations of 20 mg/mL in distilled water (PZA; Sigma-Aldrich) and 2 mg/mL in distilled water (INH and EMB; Sigma-Aldrich) or dimethyl sulfoxide (RIF; Sigma-Aldrich), filter sterilized and frozen until used.

### Broth microdilution method

Antibiotic testing in *M. marinum* was carried out in 7H9 broth by the standard microdilution method. In summary, within a 96-well plate, antibiotics were serially diluted in sterile broth media to a final concentration of 100 μL per well. To each well, 100 μL of diluted 72-hour *M. marinum* broth culture was added, yielding a final concentration of 1 × 10^5^ CFU per well in a volume of 0.2 mL. The plates were incubated at 30°C for 48 h. Following each incubation time interval, bacterial suspensions were mixed by pipetting, and a 20 μL sample was removed and serially diluted in PBS. The PBS aliquots were removed in triplicate and directly plated onto Middlebrook 7H10 or 7H11 agar media, which were sealed with permeable tape to prevent contamination and incubated for 1–2 weeks at 30°C. Experiments were repeated three times, producing results in agreement among the biological replicates. The results for each experiment for each drug combination and NP formulation are shown with the corresponding standard deviation.

### Cells and culture conditions

The human monocytic cell line THP-1 was cultured in RPMI 1640 medium with 10% (v/v) heat-inactivated fetal bovine serum. Suspension cultures of THP-1 cells were maintained at a cell density of between 5 × 10^5^ and 1 × 10^6^ cells/mL of cell culture medium and split twice per week. THP-1 cells growing in suspension were harvested at a density of 1 × 10^6^ cells/mL, resuspended in fresh medium supplemented with 5 nM phorbol myristic acid (PMA) to differentiate adherent monocytes from other cell types, and placed into 96-well tissue culture plates or 24-well tissue (Costar) culture plates containing a sterile #1 glass coverslip in each well. After overnight culture in the presence of PMA, adherent cells were washed three times gently with phosphate-buffered saline (PBS; pH 7.4) and incubated for an additional 24 h in a complete RPMI 1,640 medium with no PMA.

### Intracellular survival of *Mycobacterium*

THP-1 cells used to evaluate the intracellular survival of *M. marinum* were treated as previously described in 96-well flat-bottom tissue culture plates (Bellaire et al., 2005). Bacterial suspensions were prepared and generated by scraping 14-d cultures of the *M. marinum* grown on Middlebrook 7H10 agar into screw-cap microfuge tubes containing 7H9 media and incubated for 96 h. Pellets of bacteria were resuspended through mechanical disruption, and the numbers of bacteria present in the suspensions were determined by measuring their optical density at 600 nm (Peñuelas-Urquides et al., 2013). These suspensions were used to generate dilute mycobacteria suspensions using a complete RPMI 1,640 medium whereby the bacterial density was adjusted to the desired level to account for variations in the numbers of target monocytes. Suspensions of bacteria were added to monocyte monolayers at multiplicities of infection (bacterium:monocyte ratios) of 1:1 and 10:1. Tissue culture plates were gently agitated by hand and then centrifuged at 4°C for 10 min at 270 × g, then incubated for 90 min at 37°C with 5% CO_2_ to allow for the phagocytosis of bacteria. Monolayers were washed three times with PBS to remove any remaining non-adherent bacteria. Fresh RPMI 1,640 complete medium with 10 μg/mL gentamicin was added following the last washing. The viability of intracellular *Mycobacterium* was determined by lysing monocytes with 0.1% deoxycholate, diluting suspensions in PBS, and plating aliquots in triplicate on Middlebrook 7H10 medium. The plates were sealed and incubated for up to 14 days at 30°C. The total CFU/mL of bacteria surviving at 24 h, 48 h and 72 h were calculated based on the number of internalized bacteria detected at 2 h post-infection, representing 100% of internalized bacteria. All *in vitro* minimum inhibitory concentration (MIC) testing was conducted in accordance with the Clinical and Laboratory Standards Institute (CLSI) recommendations (Hindler and Richter, 2015). Experiments were conducted in triplicate, and the standard error is depicted on graphs unless otherwise stated.

## Results

### Antimicrobial activity of single drugs in broth cultures

To evaluate the benefits of encapsulating antibiotics in therapies against *Mycobacterium* species, NP were synthesized containing one of each of the following anti-tubercular drugs: rifampicin, isoniazid, ethambutol, and pyrazinamide. To compare equivalent amounts of the drugs, the total amount of antimicrobial present within the NP was kept equal to the total amount supplied to soluble drug-treated cultures. A fundamental benefit of NP delivery is the delayed release of the encapsulated drug; however, for the experiments described here, the absolute amount of drug present within the indicated dose of NP is equivalent to the total amount of soluble drug present. No *ad hoc* adjustments of comparisons between soluble drug and NP at each concentration were made to account for the amount of NP released or remaining encapsulated during the assay time. For example, the dose of 2 µg of soluble rifampicin is compared with NP encapsulating 2 µg of rifampicin.

**Figure 1:**
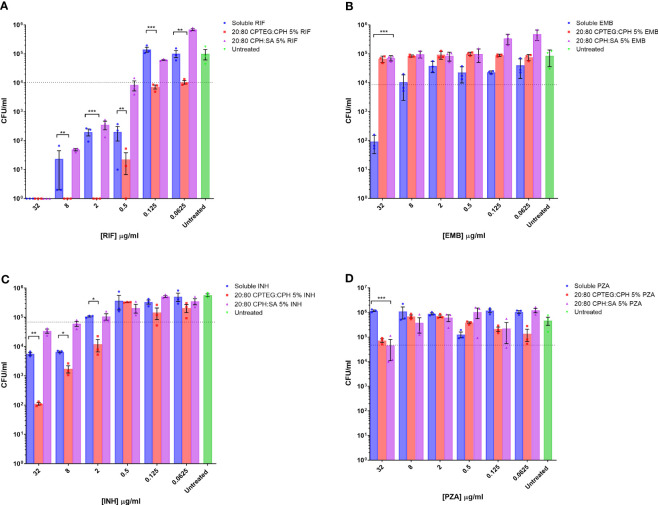
*M. marinum* drug sensitivity profiles for rifampicin and other antibiotics in broth culture media. Drug susceptibility of *M. marinum* in broth culture media after 48 h exposure to **(A)** rifampicin (RIF), **(B)** ethambutol (EMB), **(C)** isoniazid (INH), and **(D)** pyrazinamide (PZA) as a single dose of soluble drug or encapsulated in CPTEG:CPH or CPH:SA nanoparticles. All data are presented as colony-forming units per mL. The data shown are representative of similar experiments conducted in triplicate. The results of a Student’s *t*-test comparing values for soluble drugs at each concentration to nanoparticle values are indicated for corresponding *p*-values (* < 0.1, ** < 0.01 and *** < 0.001).

Drug formulations were evaluated individually against broth cultures of *M. marinum*. Early log phase *M. marinum* cultures were exposed to rifampin, pyrazinamide, isoniazid, and ethambutol in a single soluble concentration or encapsulated in nanoparticles. Following 48 h of incubation, aliquots of cultures were serially diluted and plated on a solid agar medium to enumerate viable bacteria. Individual drug sensitivity results for single drugs tested against *M. marinum* are provided in detail in **Figure 1**.

Bacterial counts from untreated cells were used to set the limit representing the inhibition of 90% of *M. marinum* (MIC_90_). The calculated MIC_90_ concentrations for the four soluble drugs fell within the expected ranges of 2 μg/mL for rifampicin (**Figure 1A**), 20 μg/mL for ethambutol (**Figure 1B**), and 5 μg/mL for isoniazid (**Figure 1C**). *M. marinum* is inherently resistant to pyrazinamide owing to poor membrane diffusion due to the unique membrane permeability of this species. Consistent with this expectation, we observed no sensitivity to soluble pyrazinamide up to the maximum concentration tested, which was 32 μg (**Figure 1D**). For isoniazid-treated cultures, the 20:80 CPTEG : CPH NP reduced *M. marinum* viability to a lesser extent than soluble isoniazid at the highest drug concentrations without changing the MIC_90_. Cultures treated with pyrazinamide NP were inhibited at the highest dose of 32 μg/mL, but soluble pyrazinamide had no effect. Rifampicin-treated cultures demonstrated higher antimicrobial activity when the drug was encapsulated in the 20:80 CPTEG : CPH NP, which reduced the MIC_90_ eight-fold compared with soluble rifampicin, from 0.5 μg/mL to 0.0625 μg/mL (**Figure 1A**). A striking finding was the absence of viable *M. marinum* in cultures treated with 20:80 CPTEG : CPH containing 2 μg/mL rifampicin. The soluble drug required 16 times that amount to achieve the same result. However, encapsulation of the drug alone is insufficient, as 20:80 CPH : SA activity was similar to that of soluble rifampicin, demonstrating that encapsulation’s benefit was chemistry-dependent.

### Antimicrobial activity of dual drug-loaded nanoparticles

**Figure 2:**
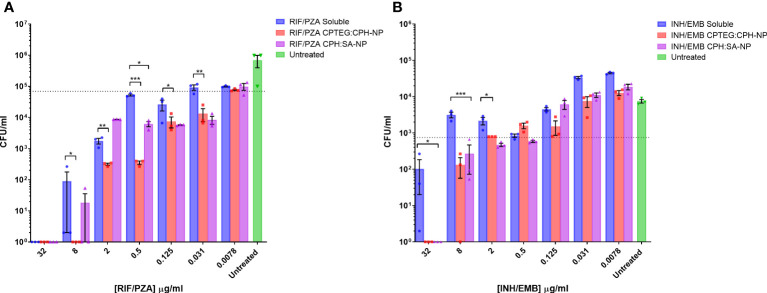
Evaluation of the antimicrobial activity of dual drug-loaded particles against *M. marinum* broth cultures. **(A)** CPTEG:CPH and CPH:SA, each with RIF/PZA. **(B)** CPTEG:CPH and CPH:SA, each with INH/EMB. Colony forming units per mL of *M. marinum* exposed to equivalent doses of soluble and nanoparticle (NP)-delivered antimicrobial compounds were enumerated at 48 h post-infection. The dotted line indicates the threshold value of MIC_90_ calculated from the average for each untreated group. The results of a Student’s *t*-test comparing the values for soluble drugs at each concentration to NP values are indicated for corresponding *p*-values (* < 0.1, ** < 0.01 and *** < 0.001).

Results from the single-drug experiments demonstrated the benefits of encapsulating anti-tubercular drugs into nanoparticles for rifampicin and isoniazid. The goal was to examine if a single NP could be synthesized to co-encapsulate the four-drug cocktail and retain antimicrobial activity (**Figure 2**). After evaluating single encapsulated drugs, we sought to co-encapsulate pairs of drugs and evaluate changes in their antimicrobial activity against *M. marinum* cultures. The randomly chosen pairs, rifampicin with pyrazinamide (RIF/PZA), and isoniazid with ethambutol (INH/EMB), were incorporated into 20:80 CPTEG : CPH and 20:80 CPH : SA. Following incubation with *M. marinum* cultures under similar conditions to the single-drug experiments, the amount of viable CFU/mL was quantified following 48-hour exposure to either soluble or equivalent amounts of drugs encapsulated in nanoparticles. The RIF/PZA cocktail encapsulated in the CPTEG : CPH NP eliminated more bacteria than a soluble cocktail of the drugs between the concentrations of 8 μg/mL and 0.031 μg/mL (**Figure 2A**), leading to a 3.47 to 900 fold increase in antimicrobial activity over this range (**Table 1**). The CPH : SA RIF/PZA cocktail did not improve antimicrobial activity to a similar degree (**Figure 2A**; **Table 1**).

**Table 1:** Fold increase in antimicrobial activity for combination drug delivery.

*In vitro* Fold Increase in Antimicrobial Activity against *M. marinum*									
ug/ml		Soluble Drug		NP-CPTEG : CPH		NP-CPH : SA		Fold Increase	
								NP-CPTEG : CPH	NP-CPH : SA
		CFU/ml	(+/- STDEV)	CFU/ml	(+/- STDEV)	CFU/ml	(+/- STDEV)		
RIF/PZA	32	0		0		0		1.00	1.00
	8	9.02E+01	(+/- 152.8)	0		1.78E+01	(+/- 30.7)	*902.33*	*5.06*
	2	1.78E+03	(+/- 630.0)	3.16E+02	(+/- 42.8)	8.71E+03	(+/- 76.7)	*5.63*	0.20
	0.5	5.29E+04	(+/- 6300.4)	3.56E+02	(+/- 76.9)	6.22E+03	(+/- 2036.7)	*148.75*	*8.50*
	0.125	2.62E+04	(+/- 17401.3)	7.55E+03	(+/- 5046.0)	5.73E+03	(+/- 230.9)	*3.47*	*4.57*
	0.031	9.33E+04	(+/- 31732.0)	1.33E+04	(+/- 10411.6)	8.44E+03	(+/- 4284.7)	*7.00*	*11.05*
	0.0078	9.96E+04	(+/- 6319.1)	7.73E+04	(+/- 7052.8)	9.91E+04	(+/- 41847.7)	1.29	1.00
INH/EMB	32	9.02E+01	(+/- 152.8)	1.00E-01	(+/- 1.6)	1.00E-01	(+/- 0)	*902.33*	*902.33*
	8	2.84E+03	(+/- 630.2)	1.33E+02	(+/- 133.3)	2.71E+02	(+/- 343.2)	*21.33*	*10.49*
	2	2.18E+03	(+/- 887.6)	8.00E+02	(+/- 0)	4.76E+02	(+/- 88.7)	*2.72*	*4.58*
	0.5	8.44E+02	(+/- 153.9)	1.60E+03	(+/- 480.5)	5.91E+02	(+/- 68.4)	0.53	1.43
	0.125	4.53E+03	(+/- 874.6)	1.51E+03	(+/- 1118.2)	6.18E+03	(+/- 2982.5)	*3.00*	0.73
	0.031	3.38E+04	(+/- 4074.1)	7.56E+03	(+/- 4287.0)	1.11E+04	(+/- 2774.2)	*4.47*	*3.04*
	0.0078	4.49E+04	(+/- 3357.0)	1.27E+04	(+/- 3385.5)	1.87E+04	(+/- 5812.2)	*3.53*	*2.40*
PIRE	32	3.92E+02	(+/- 656.3)	0		ND		*3922.667*	ND
	3.2	1.86E+04	(+/- 10555.2)	1.78E+01	(+/- 20.3)	ND		*1043.071*	
	0.32	3.43E+05	(+/- 54993.9)	3.12E+04	(+/- 3635.0)	ND		*10.99253*	
	0.032	3.61E+05	(+/- 17039.1)	4.00E+05	(+/- 89112.2)	ND		0.903333	
	0.0032	4.22E+05	(+/- 39310.7)	4.22E+05	(+/- 32331.6)	ND		1	
	0.00032	4.60E+05	(+/- 78008.5)	4.10E+05	(+/- 35345.9)	ND		1.12205	

Fold increase was calculated for each dose by dividing the average number of colony-forming units (CFUs) for soluble drug-treated cells by the average number of CFUs for nanoparticle-treated cells. Means and standard deviations are shown for experiments conducted in triplicate. Biological replicate experiments yielded similar results. For instances where no CFUs were recovered, fold reduction was calculated using a value of 0.1 CFUs to represent the fact that less than one whole bacterium was present.

The combination of ethambutol with isoniazid improved bacterial killing at higher concentrations. NP-treated cultures had significantly fewer CFU at 32 µg/mL and 8 µg/mL than those treated with soluble drugs, resulting in a >900- and >21- fold increase in antimicrobial activity, respectively (**Table 1**). Both copolymer chemistries containing INH/EMB resulted in a similar reduction in *M*. *marinum* viability. In contrast, the RIF/PZA cocktail performed significantly better with CPTEG : CPH. Considering the consistently greater antimicrobial activity of encapsulated drugs than soluble drugs in individual drug testing and cocktail loading, the CPTEG:CPH copolymer formulation’s ability to eliminate intracellular bacteria by treating *M. marinum*-infected macrophages *in vitro* was assessed.

### Antimicrobial activity of cocktail-loaded nanoparticles against *M. marinum* in broth cultures

**Figure 3:**
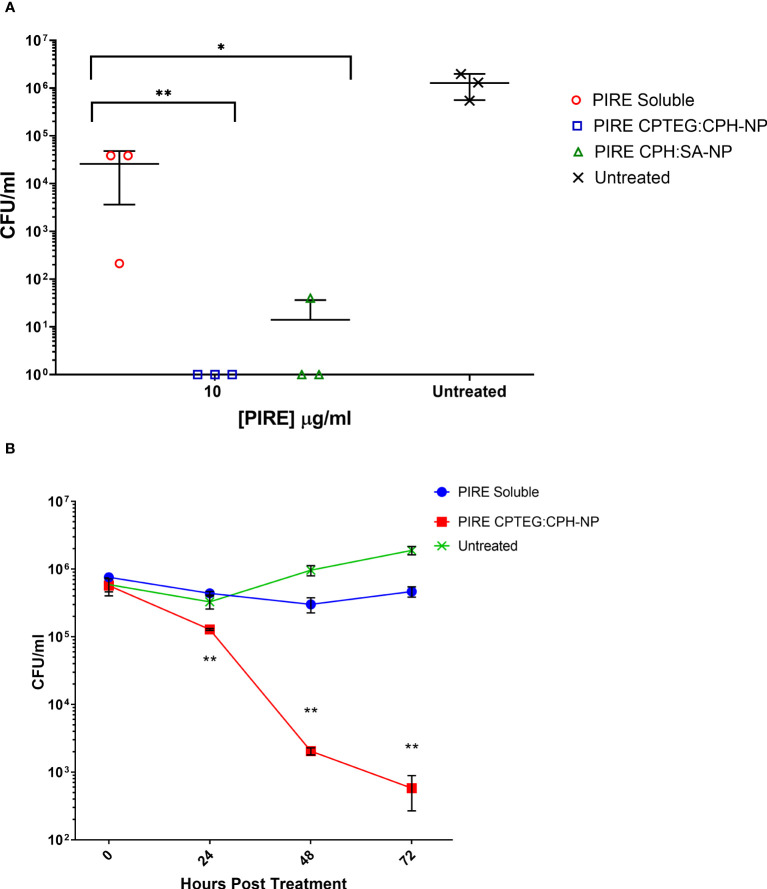
The killing of broth and intracellular *M. marinum* with rifampicin, isoniazid, pyrazinamide, and ethambutol (PIRE) CPTEG : CPH nanoparticles (NPs). **(A)** Eliminating *M. marinum* at 48 h in broth culture following treatment with equivalent doses of PIRE cocktail in soluble form or encapsulated in NPs. Delivering the drug by NP improved antimycobacterial activity significantly at a moderate dose and the highest dose, with the most significant increase in activity compared with delivery in a soluble form observed at 3.2 μg/mL. **(B)** THP-1 monocytes were infected with *M. marinum* at a multiplicity of infection of 1:1 to establish a productive intracellular infection. At 24 h, infected cultures were supplemented with 10 μg/mL of either soluble PIRE or PIRE encapsulated in polyanhydride particles. Following an additional 48 h of incubation, non-treated and drug-treated cells were washed and lysed to release intracellular bacteria. The lysate was subsequently serially diluted and plated on a solid agar medium. A significant reduction in bacterial CFU compared with the soluble drug cocktail was determined by Student’s *t*-test, and the corresponding *p*-values are shown (* < 0.1 and ** < 0.01).

All subsequent experiments examined the benefits of NP formulation 20:80 CPTEG : CPH for the delivery of PIRE, as the largest antimicrobial benefits were recorded with this formulation. The PIRE antimicrobial cocktail labeled 10 μg/mL contained 10 μg/mL of each drug, i.e., 10 μg/mL of pyrazinamide, 10 μg/mL of isoniazid, 10 μg/mL of rifampicin, and 10 μg/mL of ethambutol. Results from the *in vitro* testing of *M. marinum* broth cultures demonstrated a significant increase in antimicrobial killing (**Figure 3A**) corresponding to fold increases of >10 for 0.32 μg/mL and >3,000 for 32 μg/mL compared with soluble drugs at 48 h (**Table 1**).

We then evaluated the antimicrobial activity of the PIRE NP in human macrophages previously infected with *M. marinum*. Established intracellular infection procedures employed a multiplicity of infection (MOI) consisting of a bacteria-to-macrophage ratio at a low MOI of 1:1 and allowing the infection to establish for 24 h prior to treatment. Following the administration of 10 μg/mL PIRE in either a soluble cocktail or encapsulated in NP, the treatment was allowed to continue for 48 h before washing to remove the extracellular drug and then lysing cells to enumerate viable intracellular bacteria (**Figure 3B**). We observed that the soluble cocktail of PIRE reduced intracellular bacterial burden by 1.69 log CFU (± 1.30) compared with untreated cells. In contrast, treatment with the PIRE CPTEG : CPH NP resulted in a 6.10 log CFU reduction (± 0.32), and viable bacteria remained in the soluble treated cultures (**Figure 3B**). Surprisingly, incorporating the drugs in the 20:80 CPH : SA NP reduced the intracellular bacterial burden significantly compared with delivery as part of a soluble cocktail. The results demonstrate that delivering the PIRE cocktail within CPTEG : CPH NPs was more effective than delivering the same drugs in a soluble form, as is standard practice in healthcare, at reducing intracellular *M. marinum.*

### Kinetics of intracellular killing of *Mycobacterium*

**Figure 4:**
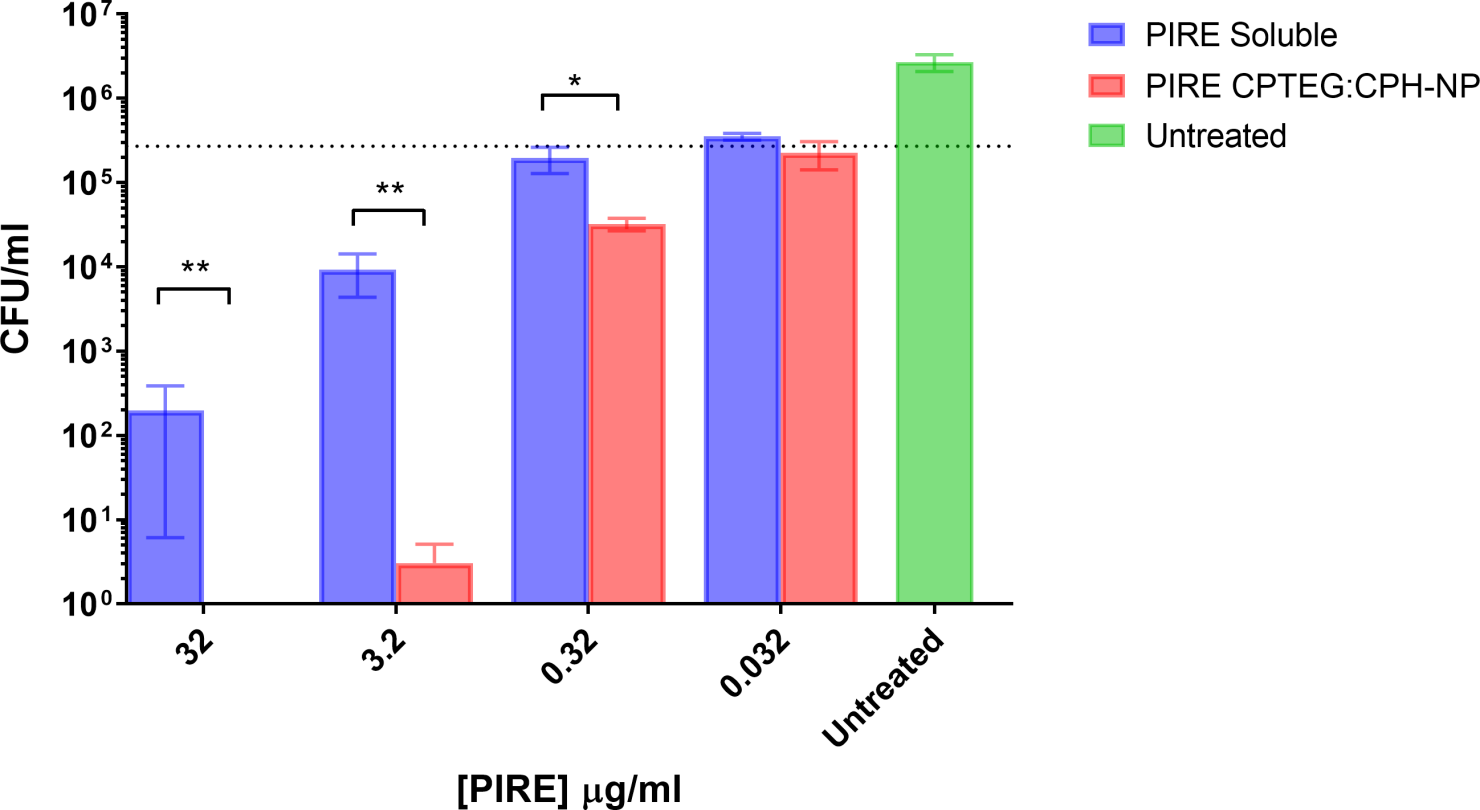
Intracellular targeting of nanoparticles (NPs) in *M. marinum*-infected macrophages. Monolayers of adherent human monocytes (THP-1 cells) were infected with *M. marinum* in a 10:1 ratio. Colony-forming units (CFU) were enumerated beginning with treatment at 48 h post-infection with either a soluble PIRE drug cocktail or an equivalent amount encapsulated within a 20:80 CPTEG:CPH NP. Intracellular bacterial viability was determined up to 72 h post-treatment, and Student’s *t*-tests were used to compare values for NP-encapsulated antimicrobials with those for soluble treatments (*p < 0.01 and **p < 0.01).

THP-1 macrophages infected with *M. marinum* at an MOI of 10:1 were treated with either soluble or NP-encapsulated drugs and followed over 72 h. At the higher MOI concentration of 10:1, the *Mycobacterium*’s survivability within macrophages increased, allowing for an evaluation of the impact of the NP-encapsulated drugs over time (**Figure 4**). Comparing the untreated cells with those treated with the 20:80 CPTEG : CPH NP formulation, the NP was better at eliminating intracellular *M. marinum*, beginning at 24 h and increasing to a maximum >3 log reduction at 72 h (**Figure 4**). At 72 h post treatment, average CFU/mL recovered from soluble drug-treated and PIRE NP-treated cells were 3.2 × 10^5^ and 6.7 × 10^2^, respectively, resulting in a calculated fold reduction greater than 1,000. At all time points, the number of mycobacteria recovered from the soluble drug-treated cells were not statistically significant compared with the untreated cells.

## Discussion

*Mycobacterium* continues to be a significant challenge to the public health community. The persistent and invasive nature of *Mycobacterium tuberculosis* models an intracellular niche layered in protection from treatment by their presence in granulomas within the deep tissue of the lower lobes of the lung alveoli. Antimicrobials that reach the bacteria must overcome the waxy mycolic acid cell wall characteristic of many mycobacteria species. Nanoparticles are capable of accepting cargo without chemically modifying encapsulated molecules, can be internalized, and persist within infected macrophages (Mullis et al., 2023). The amphiphilic nanoparticles described here improve the antibiotic efficacy *in vitro* and *in vivo* of doxycycline-based therapy against *Brucella* species, another chronic intracellular bacterial pathogen (Lueth et al., 2019). In contrast, using either rifampicin, isoniazid, pyrazinamide, or ethambutol individually is ineffective in killing intracellular mycobacteria. The 20:80 CPTEG : CPH but not the 20:80 CPH : SA NP, enhanced drug activity when directly compared with soluble formulations. The 20:80 CPTEG : CPH and 20:80 CPH : SA formulations resulted in a time- and concentration-dependent killing of rifampicin and isoniazid. Utilizing a four-drug cocktail of rifampicin, isoniazid, pyrazinamide, and ethambutol in the 20:80 CPTEG : CPH formulation provided superior benefits in drug delivery against *M. marinum.*

The *in vitro* treatment of *M. marinum* with nanoparticle formulations 20:80 CPTEG : CPH and 20:80 CPH : SA, each encapsulating the four-drug cocktail of PIRE, consistently and significantly reduced bacterial viability compared with a soluble formulation. The unique nature of mycobacteria as intracellular organisms is further enhanced when looking at their survivability in the presence of soluble drugs. We observed a noticeable reduction in the bacterial population of THP-1-infected cells for the 20:80 CPTEG : CPH and 20:80 CPH : SA formulations compared with soluble formulations, and the effect was time and concentration-dependent. This effect could be helpful in overcoming the natural resistance mechanisms of mycobacteria through colocalization and increased drug cocktail efficacy.

The use of PIRE CPTEG:CPH nanoparticles resulted in a significant reduction in viable intracellular bacteria that was not observed in the soluble drug-treated cells or those treated with the PIRE CPH:SA NP. The absence of NP colocalization with all intracellular bacteria could be investigated by incubating *M. marinum* infected macrophages with 20:80 CPTEG:CPH NP loaded only with rhodamine, as the loaded NP would eliminate bacteria if they colocalized to the same intracellular compartment. To this end, we observed that *M. marinum* in infected monocytes treated with 20:80 CPTEG:CPH nanoparticles were more likely to be within intracellular compartments associated with lysosomes. Results from this study demonstrate that amphiphilic NPs can be loaded with the four front-line anti-*Mycobacterium* drugs and serve as a promising nanotherapeutic intervention against tuberculosis.

## Conclusion

To our knowledge, this work is the first to test the effects of a polyanhydride nanoparticle against a species of *Mycobacterium*. We observed through this study the importance of developing a delivery method that can overcome the natural resistance mechanisms of mycobacteria. Delivery within a polyanhydride nanoparticle increases the efficacy of anti-tubercular drugs by increasing their intracellular delivery. The encapsulation of the four drugs into polyanhydride nanoparticles significantly improved intracellular delivery and subsequent activity against established intracellular mycobacteria. Considering these benefits, cocktail-loaded NPs represent an attractive nanotherapeutic against intrinsically difficult to treat mycobacterial infections.

## Data availability statement

The original contributions presented in the study are included in the article/supplementary material. Further inquiries can be directed to the corresponding authors.

## Author contributions

The project was conceptualized by BB and BN. Nanoparticles were designed and optimized by AM, SH, and BN. Experimental design, data analysis and interpretation were conducted by AB, BN and BB. All authors contributed intellectually to manuscript preparation, data presentation and the discussion. All authors contributed to the article and approved the submitted version.
